# Evidence-Based Quality Improvement (EBQI) in the pre-implementation phase: key steps and activities

**DOI:** 10.3389/frhs.2023.1155693

**Published:** 2023-05-24

**Authors:** Taren Swindle, Jure Baloh, Sara J. Landes, Nakita N. Lovelady, Jennifer L. Vincenzo, Alison B. Hamilton, Melissa J. Zielinski, Benjamin S. Teeter, Margaret M. Gorvine, Geoffrey M. Curran

**Affiliations:** ^1^ Department of Family and Preventive Medicine, University of Arkansas for Medical Sciences, Little Rock, AR, United States; ^2^ Center for Implementation Research, University of Arkansas for Medical Sciences, Little Rock, AR, United States; ^3^ Department of Health Policy and Management, University of Arkansas for Medical Sciences, Little Rock, AR, United States; ^4^ Behavioral Health Quality Enhancement Research Initiative (QUERI), Central Arkansas Veterans Healthcare System, Little Rock, AR, United States; ^5^ Department of Psychiatry, University of Arkansas for Medical Sciences, Little Rock, AR, United States; ^6^ Department of Health Behavior and Health Education, University of Arkansas for Medical Sciences, Little Rock, AR, United States; ^7^ Department of Physical Therapy, University of Arkansas for Medical Sciences, Fayetteville, AR, United States; ^8^ Center for the Study of Healthcare Innovation, Implementation, & Policy; VA Greater Los Angeles Healthcare System, Los Angeles, CA, United States; ^9^ Department of Psychiatry and Biobehavioral Sciences, University of California Los Angeles, Los Angeles, CA, United States; ^10^ Department of Psychological Science, University of Arkansas, Fayetteville, AR, United States; ^11^ Department of Pharmacy Practice, University of Arkansas for Medical Sciences, Little Rock, AR, United States; ^12^ Central Arkansas Veterans Healthcare System, Little Rock, AR, United States

**Keywords:** implementation science, community engagement, quality improvement, pre-implementation, implementation strategies, comparative case study

## Abstract

**Background:**

Evidence-Based Quality Improvement (EBQI) involves researchers and local partners working collaboratively to support the uptake of an evidence-based intervention (EBI). To date, EBQI has not been consistently included in community-engaged dissemination and implementation literature. The purpose of this paper is to illustrate the steps, activities, and outputs of EBQI in the pre-implementation phase.

**Methods:**

The research team applied comparative case study methods to describe key steps, activities, and outputs of EBQI across seven projects. Our approach included: (1) specification of research questions, (2) selection of cases, (3) construction of a case codebook, (4) coding of cases using the codebook, and (5) comparison of cases.

**Results:**

The cases selected included five distinct settings (e.g., correction facilities, community pharmacies), seven EBIs (e.g., nutrition promotion curriculum, cognitive processing therapy) and five unique lead authors. Case examples include both community-embedded and clinically-oriented projects. Key steps in the EBQI process included: (1) forming a local team of partners and experts, (2) prioritizing implementation determinants based on existing literature/data, (3) selecting strategies and/or adaptations in the context of key determinants, (4) specifying selected strategies/adaptations, and (5) refining strategies/adaptations. Examples of activities are included to illustrate how each step was achieved. Outputs included prioritized determinants, EBI adaptations, and implementation strategies.

**Conclusions:**

A primary contribution of our comparative case study is the delineation of various steps and activities of EBQI, which may contribute to the replicability of the EBQI process across other implementation research projects.

## 1. Introduction

Community-engaged research is “the process of working collaboratively with groups of people affiliated by geographic proximity, special interests, or similar situations concerning issues affecting their well-being”(1). The concept of engaging community partners in all aspects of research is grounded in the notion that the population impacted by the issue, condition, or situation has a unique perspective on the resolution of the issue, which is critical to ensuring the effectiveness and adequacy of health interventions in broader community settings (2, 3). Engaging community partners in health research has been proven to be significant in efforts to improve population health in areas such as diabetes, nutrition, infant mortality, cancer, obesity, dental hygiene, etc (4–8). Dissemination and implementation (D&I) science researchers began to describe the need for participatory engagement among local practitioners nearly two decades ago (9). Since that time, the field has increasingly recognized the value of involving partners to solve implementation problems and advance solutions that support equitable implementation (10, 11).

The combination of community-engaged research and D&I, termed community-engaged dissemination and implementation (CEDI) research, reflects the intersection of community-partnered research in implementation research design, methods, and dissemination (12). The overall goal of CEDI methods is to foster the translation of research findings to improve population health by the uptake of evidence-based interventions (EBIs) in communities (13). Examples of CEDI methods include implementation mapping, concept mapping, group model building, and conjoint analysis (14, 15). CEDI approaches are increasingly recognized as critical to the selection and tailoring of implementation strategies (16). Evidence-Based Quality Improvement (EBQI) (17) is another key example of a CEDI method to accomplish engagement of key community partners in the implementation process, although it has not been consistently named in CEDI or implementation literature (16, 18).

EBQI is related to but distinct from the more broadly known concept of Quality Improvement (QI). QI aims to improve local multi-level processes and outcomes by using data from the local context, local expert input and opinions, and local multi-disciplinary teams (17). A review and critical appraisal of the existing literature is not typically part of a QI process (19); thus, the addition of the term “evidence-based” to QI was made to distinguish a QI process that integrates research evidence into decisions (17). That is, EBQI expands on QI by integrating local input with the *best available research evidence* at all stages of the process, from the “diagnosis” of performance issues to the development and tailoring of implementation strategies (20), and in some cases through the process of evaluation (21). Specifically, EBQI involves implementation science researchers and local partners working as a team to adapt EBIs (i.e., programs, principles, procedures, products, policies, practices, pills) (22) and select and tailor implementation strategies designed to improve system processes for uptake of the evidence (i.e., the “how” of getting the system to use the EBI). Studies that have measured health outcomes related to the use of EBQI suggest positive effects (21).

In the literature to date, EBQI has been called a myriad of terms (21). The developers of EBQI have used terms such as method (19), multi-level approach (17), and multi-faceted implementation strategy (19). Co-authors on this paper have also referred to EBQI variably as a process, technique, and tool. Thus, the language around EBQI seems to reflect the “idiosyncratic use of … terms involving homonymy (i.e., same term has multiple meanings), synonymy (i.e., different terms have the same meanings), and instability (i.e., terms shift unpredictably over time)” (18) that has plagued implementation science in its developmental years. Drawing on an understanding of QI and EBQI's distinct features from QI, we define EBQI as a *deliberative, partnered, and evidence-driven process* to inform the selection and tailoring of implementation strategies and EBI adaptations. This definition of EBQI reflects a conceptualization that EBQI would fit under the umbrella of more global *approaches* to research (e.g., Community-Based Participatory Research, CEDI) and could be operationalized with other *methods* (e.g., network analysis, formative evaluation). We acknowledge that EBQI can be applied across all stages of implementation (20) and that engagement of community partners and key interested parties is critical at all stages of implementation. However, our attention in this perspective is more narrowly focused on the pre-implementation phase. Pre-implementation is a critical phase where key decisions are made, and input and engagement from various partners is critical for addressing contextual conditions and improving implementation success.

To date, steps in the EBQI process have included (1) the formation of local teams to consider data on barriers and facilitators to implementation and (2) drafting, iterating, and planning a locally contextualized implementation strategy to increase uptake of an EBI (20). Additionally, EBQI activities have been described as: stakeholder planning meetings using expert panel techniques to identify priorities, formative evaluation, development and training of local QI champion and team members, practice facilitation, and review of local QI proposals (5); monthly calls to facilitate collaboration and spread of EBIs; and technical work groups to support local priorities for EBIs (23). A recent scoping review of EBQI found the most common components across 211 studies to be: use of research to select effective interventions, engagement of stakeholders (i.e., partners), iterative development, partnering with frontline implementers, and data driven evaluation (21). This illustrates variety in application of EBQI in the extant literature.

The purpose of this paper is to illustrate the steps, activities, and outputs of EBQI in the pre-implementation phase as operationalized across seven projects to illustrate common elements and variations in application of EBQI (20). This goes beyond the recent scoping review (21) to provide specifics of key case examples that illustrate common and replicable processes of EBQI. Steps are defined as components in the EBQI process; activities are the methods used to achieve those steps (20). This paper will focus specifically on the use of EBQI in the pre-implementation phase to select and tailor strategies and/or adapt EBIs. In so doing, this paper provides a multi-disciplinary exposition of the application of EBQI for advancing implementation initiatives across diverse service contexts, examining the following research questions:

(1) What steps do researchers accomplish using EBQI in practice?
(2) How do researchers accomplish the steps of EBQI? That is, what activities are used to accomplish EBQI steps?

## 2. Case selection

To identify key steps, activities, and outcomes of EBQI methods, we retrospectively examined a set of seven case examples of EBQI application in research projects. Specifically, our goal was to use case examples to create a holistic description of EBQI and capture how each case selected and tailored implementation strategies and/or made EBI adaptations that would be subsequently tested in a research study (24). We applied steps of comparative case study methods to achieve this goal including: (1) specification of research questions, (2) selection of cases, (3) construction of a case codebook, (4) coding of cases using the codebook, and (5) comparison of cases (21).

Natural variation and overlap in the cases were a key interest. Specially, cases were purposively included to maximize variation (24) in the EBIs to be implemented, contexts for implementation, and processes of engagement applied across known users of EBQI in our networks. Inclusion criteria for cases included: (1) explicit claim of application of EBQI processes, (2) engagement of community or clinical partners in EBQI process, (3) targeted outcome of selecting and tailoring implementation strategies or EBI adaptations through EBQI, and (4) representation of funded research among the author group. All included cases were part of IRB-approved studies from our respective institutions.

### 2.1. Case codebook

The research team developed a case codebook to collect a standard set of information for each case and coded each case using this codebook. This codebook included basic features of the EBQI process (e.g., number and modality of meetings, partners engaged), the progression of EBQI meetings, and activities that were used at each meeting. Using the codebook, the lead investigator for each case extracted details of their respective projects. When needed, the lead author solicited additional information or clarification from investigators. This directed template analysis approach (25) allowed for focus on the study elements most meaningful for comparison. Additionally, one study provided a meeting-by-meeting description of the EBQI process to provide greater detail on the activities of each meeting and provide illustrative examples. After extraction of this information, lead investigators on each case example met to discuss commonalities and differences across cases as well as the progression and activities of each case.

## 3. Case comparison

The team completed a cross-case analysis to identify similarities, differences, and the range of steps, activities, and outputs across cases. We used this comparison to generate a list of the key steps of EBQI and corresponding examples of activities to accomplish each step. Table 1 details the targeted EBIs and contexts for implementation as well as steps, activities, partners involved, and outputs of the 7 case examples. The selected cases included 7 distinct settings (e.g., early care and education, community correction centers, hospitals), 7 EBIs (e.g., cognitive processing therapy, violence prevention program, exercise program) and 5 unique lead authors. Cases examples include both community-embedded and clinically-oriented projects. For example, Teeter and colleagues (30) deployed EBQI to adapt a pharmacist-initiated intervention for naloxone in community pharmacies, while Zielinski et al. (29) used EBQI to prioritize determinants, identify implementation strategies, and create an implementation plan for supporting uptake of cognitive processing therapy in prisons.

**Table 1:** Case description template.

Author	Evidence-based intervention (EBI)	Context	# of Meetings	Steps^a^	Partners involved	Modality of engagement	Products/Outcomes
Swindle (26)	De-implementation of detrimental feeding practices	Early Care and Education	5	1, 2, 3, 4, 5	Teachers, parents, directors in LA; 2 EBQI peer mentors from AR	Primarily in-person with some partners joining remotely for some sessions	Package of 5 strategies
Swindle (27)	Exercise intervention for expecting women with excess weight	Community	5	1, 2, 3, 4, 5, 6	WIC, parks and Recreation, Insurance, faith leaders, trainers, mental health, mothers	Shifted to virtual after 1 session because of COVID-19	4 key adaptations and 3 implementation strategies
Lovelady (28)	Hospital-based Violence Intervention Program	Hospital & Community	2	1, 2, 3, 4	Medical providers, patients, social service orgs	In-person with one person joining *via* zoom during one session	Top 8 Barriers, Top 3 Facilitators, and Strategies for each
Zielinski (29)	Cognitive Processing Therapy, adapted for prisons (CPT-CJ)	Correction Centers	4	1, 2, 3, 4, 6	Correction center counselors, administrators, and security staff	Virtual	List of anticipated barriers and facilitators that were used to adapt materials & implementation plan; approved implementation plan
Teeter (30)	Pharmacist-initiated intervention for Prescribing and Dispensing Naloxone	Community pharmacies	4	1, 2, 3, 4, 5	Pharmacy district manager, pharmacists, community informants/patients, pharmacists’ association representative	In-Person	Patient-facing strategies to engage and educate; Pharmacist-facing strategies to train and educate, adapted to changing infrastructure
Synder/Curran (31)	Patient-reported outcomes (PROs) in community pharmacies	Community pharmacies	5	1, 2, 3, 4, 5	Pharmacist owners/pharmacists, pharmacy staff, patients	Virtual	Adapted PRO process and package of implementation strategies “ready to pilot”
Fortney/Curran (32)	Mental health EBPs for treatment-resistant depression/bi-polar disorder/risky drinking	FQHCs	Variable across 2 projects, minimum of 4	1,2, 3, 4, 5, 6	Site clinicians (physicians and nurses), patient representatives, EBP experts	Telephone	Selected and adapted EBPs and implementation strategies

^a^ 1. Form a team of local partners; 2 Prioritize determinants; 3. Select EBI adaptations/implementation strategies; 4. Specify EBI adaptions/strategies; 5. Refine EBI adaptations/strategies; 6. Make research design decisions.

Cases were examined and compared for key basic features including the number of EBQI meetings held, types of partners included, and modality of meetings. Included case studies ranged in the number of meetings from 2 to 5 (26, 27, 31). On average, included cases were 4 meetings long (Median = 4). EBQI processes with greater number of meetings were observed for projects that included selecting and tailoring both adaptations and implementations, whereas projects targeting more discrete pre-implementation tasks (e.g., prioritizing determinants) met objectives in fewer meetings. Case examples included between three and seven partner sectors in the EBQI meetings (Mean = 4, Median = 3). Most projects included partners across different levels of implementation (e.g., front line implementer, leader, end user). Most (6/7) cases included end users in the process (i.e., patients, parents). The modality of meetings across cases included one example that was fully in-person; three examples that were fully virtual/remote; and 4 that included a mix of in-person and virtual strategies.

Commonalities and variations across case studies suggest basic steps that are core to EBQI in the pre-implementation phase; these are presented in Table 2 and include: (1) forming local teams of partners and experts, (2) prioritizing determinants, (3) selecting EBI adaptations and/or implementation strategies, (4) specifying and tailoring selected adaptations or implementation strategies and (5) refining EBI adaptations and/or implementation strategies. Most (7/8) cases included all these steps; three included an additional step of making research design decisions (e.g., choosing the control condition; selecting/refining measures). For the third step of selecting EBI adaptations and/or implementation strategies, all cases selected implementation strategies, while 3 also selected adaptations of the EBI (27, 29, 34).

**Table 2:** Activities for each EBQI step.

Steps	Activities
1. Form a team of local partners and experts.	Nomination by key informants (22–25, 28, 33); Nomination by study partners (21, 23, 33); Sector based recruitment (24, 28); Random selection from study sample (28); Inclusion of target population (21, 23–25, 33); Goal Setting (21)
2. Prioritize determinants.	Card sorting (24), Provide numeric rankings (22, 23, 28); Presentation/discussion of interview findings (23, 28, 33); Online individual brainstorming (28); Review previous research and have guided discussion (21, 23, 33)
3. Select EBI adaptations/implementation strategies.	Concept Mapping (24, 25); Live-edit documents during presentation of previous research and guided discussion (22, 28); Online individual brainstorming (21, 28); Nominal Group Technique (33); Consensus discussion with voting (33)
4. Specify and tailor EBI adaptations/strategies.	Liberating Structures (24, 25); Nominal Group Technique (23, 25); Live-edit documents during guided discussion (22, 28); Present suggestions based on prioritized determinants and gauge reactions (21, 33)
5. Refine EBI adaptations/strategies.	Breakout rooms with discussion questions (24); Chat probes (22, 24); Liberating Structures (24, 25); Complete implementation planning guide (22); Live-editing, Presentation of previous research and guided discussion, and group consensus (21, 28); Presentation of mid-pilot outcomes and guided discussion (22, 28)

The activities taken to achieve these steps were diverse (See Table 2). Each step had between 4 and 6 unique activities identified (Mean = 4) Commonly, cases included nomination of key informants and rapport building exercises in the first step of forming a team. Numeric rankings and guided discussions were common activities for the second step of prioritizing determinants. For the third step of selecting adaptation and implementation strategies, brainstorming and seeking consensus were common. Presenting ideas to gauge reactions and the nominal group technique were used in multiple cases for the fourth step of specifying and tailoring strategies. Finally, guided discussion of mid-point results was the most prominent activity of refining/iterating adaptations and implementation strategies. Some activities were used across multiple steps [e.g., Liberating Structures (https://www.liberatingstructures.com/), live editing of documents], illustrating the flexibility of activities to achieve multiple purposes.

We have expanded on the Swindle (27) case to provide a meeting-by-meeting description of an EBQI process for the entirety of the pre-implementation phase, from launch to preparation for implementation. This includes the steps, activities, and outcomes of each individual meeting. (See Supplementary). The project described in this case study was designed to adapt a clinical exercise intervention for expecting women with excess weight for community-based delivery and establish a starting point for implementation strategies in the new setting. This process resulted in 3 key EBI adaptations (hybrid delivery, refined incentives, and post-partum support) and 3 implementation strategies (community-academic partnerships, centralized technical assistance, and involving participants' family/social support).

## 4. Recommendations for EBQI in the pre-implementation phase reflecting our case comparison

This perspective examined 7 case studies of the application of EBQI in the pre-implementation phase. Comparison of cases suggested 5 common steps of EBQI to prepare for implementation. These steps cut across the variety of settings and EBIs included in our case examples, which illustrates the widespread applicability of these steps. For each step, we identified several activities. That is, various activities were used across the cases to achieve each step. The diversity of activities identified illustrates how each step may be achieved depending on the context and needs of the project. Commonly, the steps identified led to prioritized determinants of implementation, adaptations for EBIs, and fully specified implementation strategies ready for testing. As such, the primary contribution of our perspective is the delineation of steps and activities of EBQI, particularly when used as a deliberative, partnered, and evidence-driven process to inform the selection and tailoring of implementation strategies and EBI adaptations prior to implementation. Thus, our work answers a recent call to provide transparency and detailed descriptions for the process of tailoring in implementation science (33).

Ultimately, the process of EBQI identified in included cases expands on steps of the EBQI model as laid out by early users (20). The 5 common steps identified were: (1) forming a local team of partners and experts, (2) prioritizing implementation barriers and facilitators (i.e., determinants) based on existing literature/data, (3) selecting and tailoring implementation strategies and/or EBI adaptations in the context of key determinants, (4) specifying selected strategies/adaptations, and (5) refining strategies/adaptations. These 5 steps tease apart and add detail to the 2 steps advanced by Curran and colleagues in 2008 (20). Notably, only some (3) of our cases included adaptations to the EBQI. We recommend the decision to adapt an EBI be driven by the prioritized determinants of implementation. That is, when fit of the EBI with the context is a barrier, adaptation is likely needed. Further, some cases involved a sixth step of making research design decisions (e.g., refining focus group questions, choosing control group, selecting measures). Each of these steps helps to prepare for a local implementation effort. Consistent with implementation science theory (28) and the spirit of CEDI (14), we view the emphasis on local knowledge and expertise as particularly important and recommend that considerations for selecting, tailoring, and iterating adaptations and strategies be made if the EBI or implementation strategy is transferred to another context. That is, by design, the ideas and priorities from one EBQI process may or may not translate to other settings with different contextual considerations.

Within each EBQI step, we identified several activities. This illustrates a non-exhaustive catalogue of options for *how* to move through EBQI in the pre-implementation phase. Key to many of our activities and an important recommendation for future application of EBQI is the inclusion of end users, which was present in 6 of our 7 cases. Other authors have made a compelling case for the importance of participatory approaches for optimizing fit of EBIs within context (35), addressing structural racism (11), and advancing equity (36). Our cases illustrate options for structuring input and balancing power with other types of partners. We acknowledge that power balance with end users (e.g., patients) and implementers (e.g., physicians) is not always possible, and some groups may choose to conduct parallel EBQI processes with implementing partners and end users as in our Snyder/Curran case study (31).

Notably, EBQI has historically been and continues to be used beyond the pre-implementation phase. Work by Hamilton and colleagues (23) illustrates that EBQI can function as an implementation strategy during the process of implementation rather than a time-limited process that ends when implementation begins. In fact, EBQI may be a “meta-strategy” during the active implementation phase through which many other strategies can be decided upon and deployed (e.g., working groups, facilitation calls, champion engagement). We believe operationalizing EBQI as an implementation strategy is most fitting when the purpose is “to enhance the adoption, implementation, and sustainability of a clinical program or practice” (37) or for “creating buy-in among stakeholders.” (34) The Department of Veterans Affairs (VA) Quality Enhancement Research Initiative (QUERI) implementation road map (38) conceives of EBQI in this way and illustrates how EBQI operates during both active implementation and sustainment phases. Continuation of EBQI engagement across implementation phases allows continuation of partnerships formed in pre-implementation. Some cases included in our comparison reconvened EBQI panels after pilot tests to inform further refinement of implementation strategies and research designs as well as community expansion (27). Consistent with prior literature (14), we believe partner engagement is critical in and beyond the pre-implementation phase. Thus, this perspective specifies the steps and activities of a specialized use of EBQI. This example may be useful for specifying steps and activities of EBQI across all phases of implementation.

We believe EBQI used at any phase of implementation is an example of quality CEDI work and fits with other recommended CEDI methods (12). However, we acknowledge this perspective is limited by over representation from one academic institution's understanding and application of EBQI. Our delineation of the steps and activities of EBQI for pre-implementation provides a basis from which others can compare and contrast their use of EBQI and other CEDI approaches and methods (e.g., implementation mapping, group model building). One promising way to advance this work is conceptualizing these CEDI approaches and methods as complex interventions and studying them using the lens and methods of functions and forms (39). Future work on EBQI can expand on both steps (e.g., which steps are pursued always vs. as-needed; which additional steps need to be considered) and activities to fulfill those steps, including developing tools and guidance for when and how to apply each step for maximum benefit. Future research may also compare EBQI as a process for selecting implementation adaptations and strategies to alternative processes (e.g., Implementation Mapping) to identify potential differences in the effectiveness of the outputs and/or partners' satisfaction with the process.

## Data Availability

The original contributions presented in the study are included in the article/**Supplementary Material**, further inquiries can be directed to the corresponding author.
