# Supplementary material for: Evidence-Based Quality Improvement (EBQI) in the pre-implementation phase: key steps and activities
Source: Front Health Serv. 2023 May 24;3:1155693. doi: 10.3389/frhs.2023.1155693 (PMC10244502; doi:10.3389/frhs.2023.1155693)
Supplement: Supplementary file 1 [file Table1.docx]

Supplement: Expanded Case Study Session Description

| **EBQI Meeting** | **Functions** | **Forms** | **Outcomes** |
| --- | --- | --- | --- |
| 1. January 2020   (In person) | - Forming a local team of partners and experts - Prioritizing implementation determinants | - Solicit feedback on Memorandum of Understanding. - Provide overview of project and preliminary interview results (N=6 complete). - In groups of 2-3, brainstorm barriers/facilitators/ideas on colored sticky notes to be clustered together on the walls around the room for discussion. - Assigned homework of bringing back the names of persons/places we should know to support this work. | - Revised MOU - Identified key places, community resources, and potential mechanisms for success. Documented logistics and practical considerations likely to threaten success. - Acknowledged what panel members and their networks brought to the effort. - Determined need to recruit insurance representative to the panel. |
| 1. April 2020   (virtual due to COVID-19) | - Prioritizing implementation determinants - Make research design decisions | - Gather feedback and set priorities on interview data (N =8 completed). - Round robin sharing of names/places to know to support this work. - Individual time to process the executive summary of interviews/focus groups to date and in breakout rooms discuss (a) 2 things they found interesting or unexpected (b) 1 thing that moves forward or changes understanding of the problem. - Advise on remaining focus groups (N = 1 complete); Invited to unmute and share or chat to share probes for remaining interviews/focus groups. - Fill in the blank in chat: “Based on our work together today, it feels important that we….” - Assigned homework to email the research team one thing that went well and one thing to improve on the virtual format. | - Generated list of 22 names and 15 places to support the work in Arkansas. - Identified that panelists found preliminary findings on locations and incentives as most interesting; findings on workout partners as important to advance understanding. - Added probes to interviews/focus groups to probe for top barrier, preferred locations, additional resources requested, why peer support was important, and probe about post-delivery exercise. - Gathered feedback on virtual format including preference for use of breakout rooms. |
| 1. May 2020   (virtual) | - Selecting strategies and adaptations in the context of key determinants, | - Fill in the blank, “Life in quarantine is…” - Share and discuss final interview and focus group results. - Break into 4 breakout rooms and generate 3 ideas that could work in the real world for an assigned topic (e.g., recruiting, incentivizing, selecting locations). Small groups share back to large group who asks questions, gives reactions, and denotes favorite ideas. - Assigned homework of web survey rating the importance and feasibility of generated ideas (1 = Not Important, 10 = Extremely Important). | - Generated 33 ideas for community delivery for feasibility and important ratings across 8 key topic areas. |
| 1. July 2020   (virtual) | - Specifying selected strategies and adaptations | - Share in chat, “How are back to school decisions impacting you and your work?” - Break into 4 breakout rooms to discuss similarities and differences between partner and research ratings (visuals provided). Discuss and select top two ideas that balance perspectives. Define selected ideas (e.g., who would do what, what resources would be needed). Small groups share back to large group on selections for feedback. - Develop consensus for top to strategies per topic area to prioritize pilot | - Developed consensus around 3 important adaptations and 3 implementation strategies for community delivery. |
| 1. November 2020   (virtual) | - Refining strategies and adaptations | - Share in chat, “What’s the biggest news in your world since we were together last?” - Researchers presented brief overview of 3 adaptations and 3 implementation strategies. Community partners selected which they wanted to discuss in-depth and joined breakout rooms for each. Researchers solicited general input and posed specific questions to refine. Rejoined large group to debrief general reactions and potential blind spots. - Researchers shared draft of visual/branding ideas and asked for reactions and input to refine. | - Incorporated feedback to fully specify prioritized adaptations and strategies using the AACT framework. - Solidified visual branding concept. |
